# Spontaneous Distal Ureteric Rupture in a Young Patient With Fragile X Syndrome

**DOI:** 10.7759/cureus.78666

**Published:** 2025-02-07

**Authors:** Haadia Safdar, Mecaelan Sardar, Fuad Shaukat, Ammar Sarwar Abbasi, Maria Tunio

**Affiliations:** 1 Urology, Medway NHS Foundation Trust, Gillingham, GBR; 2 Emergency Department, University Hospitals Plymouth NHS Trust, Plymouth, GBR

**Keywords:** fragile x mental retardation 1, fragile x syndrome, proximal ureter stone, spontaneous ureteric rupture, ureter rupture

## Abstract

This report describes the case of a patient in her late teens with fragile X syndrome, developmental delay, and recurrent urinary tract infections who presented to the emergency department with a productive cough, weight loss, and being generally unwell over the past few weeks. She was found to have a firm, distended abdomen and, while being investigated for sepsis of unknown source, deteriorated rapidly and was intubated and ventilated in the intensive care unit (ICU). After multiple imaging studies, she was diagnosed with left ureteric rupture secondary to a left distal ureteric calculus, resulting in a urinoma in the left retroperitoneal space. An interventional radiology-guided drain was inserted to drain the urinoma, and a left nephrostomy and anterograde ureteric stent were inserted. Her condition improved after these interventions, and she was later extubated and discharged from the ICU to the general ward.

## Introduction

Spontaneous ureteric rupture is a rare condition in urology, with its underlying mechanism often poorly understood. One of the most common causes is the presence of ureteric stones, as demonstrated in this case. Other potential causes include malignancy, bladder outlet obstruction, idiopathic retroperitoneal fibrosis, and certain connective tissue disorders [1]. Due to its non-specific symptoms, spontaneous ureteric rupture is frequently mistaken for other intra-abdominal pathologies, which can lead to delays in diagnosis and treatment, potentially worsening patient outcomes. Therefore, a high clinical index of suspicion is essential. The diagnosis is typically confirmed through imaging, with CT urogram being the first-line modality [2]. Management should be tailored to the individual patient, considering the underlying etiology, clinical stability, and presence of complications. Treatment options include conservative management, interventional procedures, or open surgery, depending on the specific circumstances [3].

## Case presentation

A female in her late teens, with a history of fragile X syndrome and developmental delay (non-verbal), presented to the emergency department (ED) accompanied by her mother, who served as her primary carer. The mother reported that the patient had been experiencing a productive cough with green sputum, weight loss, and general malaise for the past few weeks. During this period, the patient had a significant loss of appetite, resulting in notable weight loss. She also became increasingly lethargic and had reduced mobility, no longer able to walk or bear weight at home. Additionally, the patient had a history of recurrent urinary tract infections, for which she had received multiple courses of antibiotics. A urine culture conducted a month prior had grown an extended-spectrum beta-lactamase-positive organism. The patient also had baseline bladder and bowel incontinence.

Upon presentation to the ED, the patient was found to be hypotensive, with a systolic blood pressure of 76 mmHg. A venous blood gas revealed metabolic acidosis and a raised lactate of 6.0 mmol/L (normal: <2 mmol/L). Given the elevated lactate, the patient was escalated to the critical care team and transferred to the high-dependency unit with a working diagnosis of sepsis of unknown origin. Overnight, the patient’s respiratory distress worsened, the metabolic acidosis persisted, and her Glasgow Coma Scale (GCS) score dropped to less than 8 (normal GCS score: 15/15), necessitating intubation and ventilation. She was subsequently transferred to the intensive care unit (ICU), where a noradrenaline infusion was initiated to stabilize her mean arterial pressure.

On physical examination, a distended abdomen with a firm mass in the left upper quadrant extending to the epigastric region was noted. Laboratory results (as shown in Table 1) revealed raised inflammatory markers. The patient was started on broad-spectrum intravenous antibiotics (piperacillin-tazobactam and clarithromycin, later switched to meropenem).

**Table 1:** Blood results showing raised inflammatory markers.

Parameters	Value	Normal range
Procalcitonin	524 ng/mL	<0.05 ng/mL
C-reactive protein	313 mg/L	<10 mg/L
White cell count	24,000 cells/µL	4,500–11,000 cells/µL
Lactate	9.5 mmol/L	<2 mmol/L

**Figure 1:**
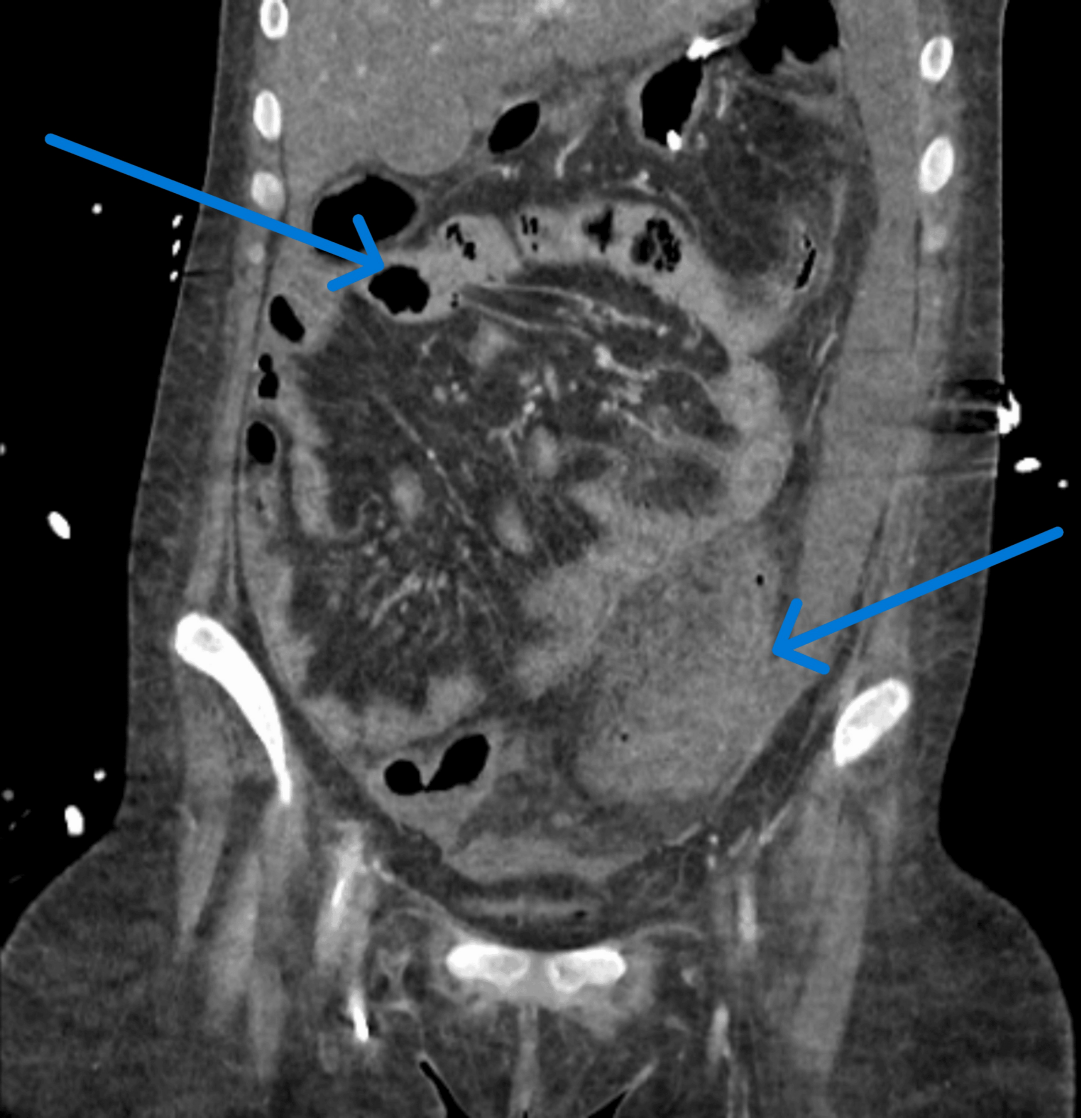
Contrast-enhanced CT scan of the abdomen and pelvis suggestive of inflammatory bowel disease involving most of the colon with ascites, along with adjacent inflammation of the left psoas muscle.

An initial contrast-enhanced CT of the abdomen and pelvis (Figure 1) raised the possibility of colitis or inflammatory bowel disease (IBD). However, the patient’s clinical symptoms did not align with IBD. Gastroenterology was consulted and recommended further investigation, noting that other inflammatory processes could mimic an IBD-like picture on CT.

**Figure 2:**
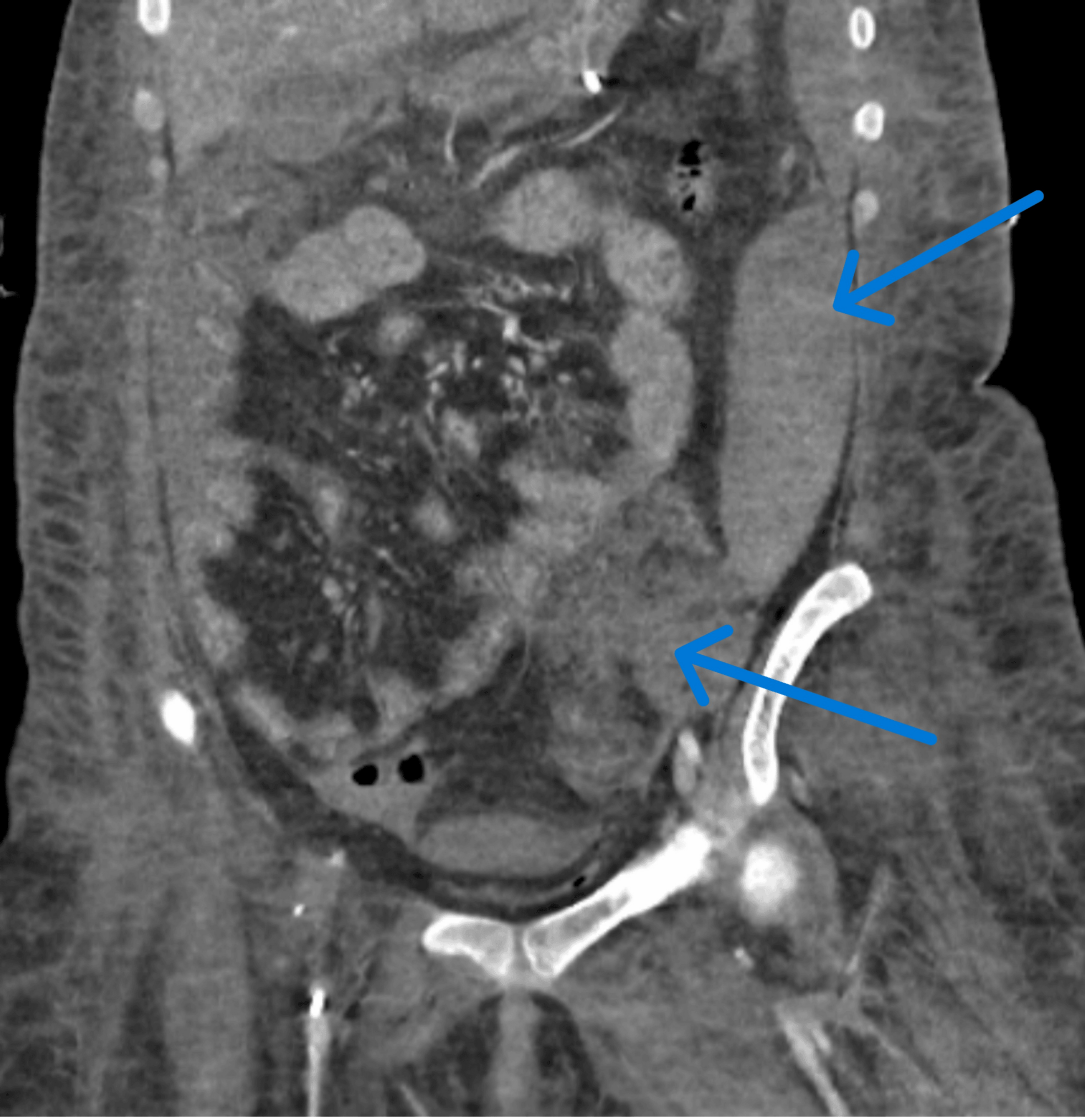
Contrast-enhanced CT of the abdomen and pelvis revealing diffuse swelling of the left iliopsoas muscle showing linear hyperdensities, suggesting an active bleed which is seen extending medially to form a loculated hyperdense collection in presacral region.

A subsequent CT scan (Figure 2) revealed evidence of an intra-abdominal hemorrhage with moderate intraperitoneal fluid accumulation, thought to be a psoas muscle hematoma. The patient was taken to interventional radiology (IR) for embolization of a suspected hemorrhaging vessel, but no hemorrhage was found, and no intervention was performed.

**Figure 3:**
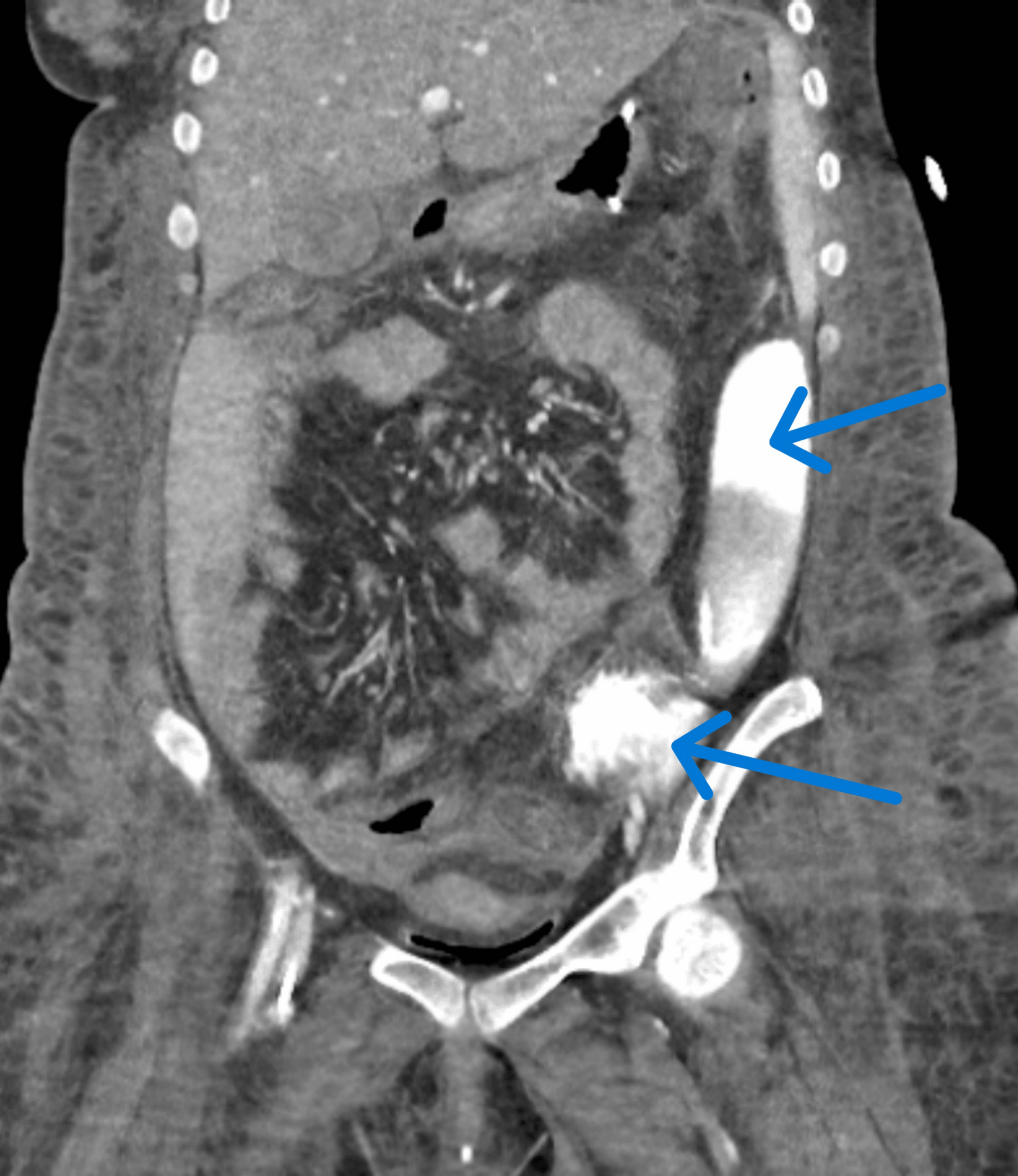
Contrast-enhanced CT of the abdomen and pelvis suspicious for a left distal ureteric calculus, resulting in the rupture/perforation of the left ureter in the left iliac fossa with resulting urinoma in the left retroperitoneal space, eventually extending into the peritoneal space in the left iliac fossa.

A third CT scan (Figure 3) conducted the following day identified a left distal ureteric calculus, which had caused rupture of the left ureter in the left iliac fossa, resulting in a urinoma in the left retroperitoneal space.

**Figure 4:**
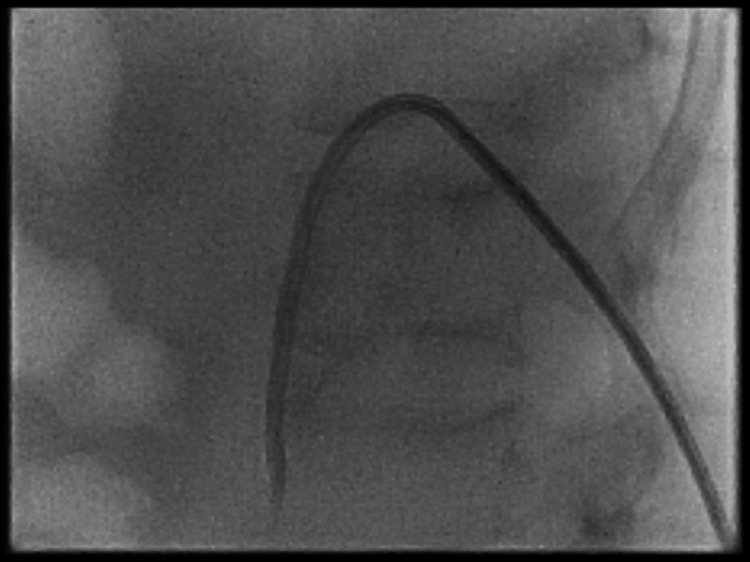
Fluoroscopy showing left nephrostomy. An 8-French locking pigtail nephrostomy was inserted and urine was noted to be draining freely.

The management of the patient was initially challenging due to the lack of a definitive diagnosis. Treatment focused on supporting her hemodynamics and ventilation in the ICU until the diagnosis was confirmed. Once the urinoma was diagnosed, an IR-guided drain was placed to drain the collection. The following day, a left nephrostomy (Figure 4) was inserted, which began draining frank blood. This helped reduce the elevated intra-abdominal pressure, allowing for successful extubation. The abdominal drain fluid cultured *Bacteroides fragilis*, which was sensitive to meropenem, so her antibiotic regimen was continued with the addition of metronidazole. The abdominal drain was removed a few days later. A nephrostogram confirmed that the nephrostomy was correctly placed, with free contrast flow through the ureter and no evidence of contrast extravasation.

**Figure 5:**
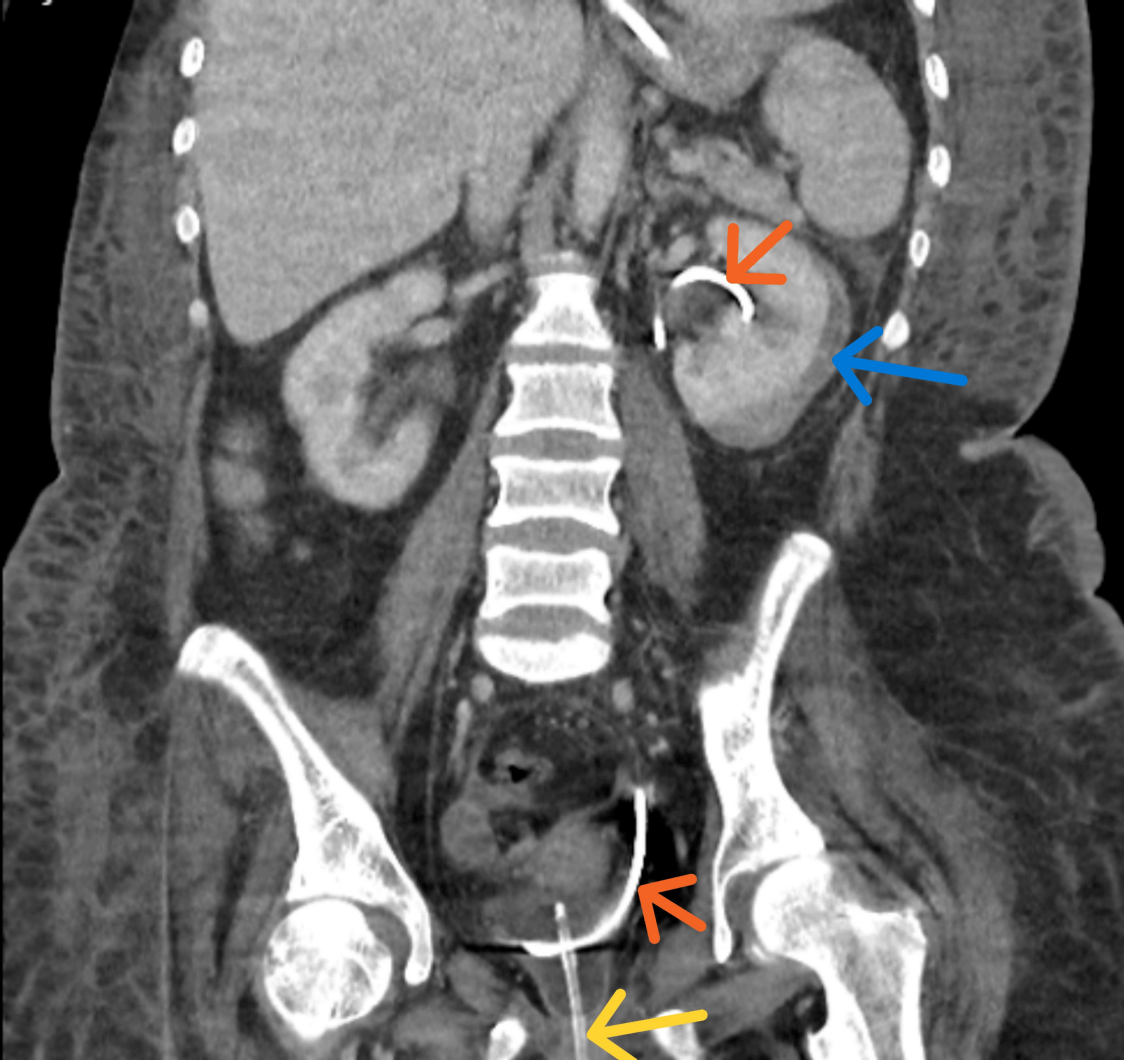
CT of the urinary tract showing a left-sided ureteric stent in situ (orange arrow) and urinary catheter in situ (yellow arrow). Improvement in the left retroperitoneal, pelvic, and abdominal collection was noted. No new collections were identified. Stable appearances of the left kidney subcapsular collection were noted (blue arrow).

Shortly thereafter, a left anterograde ureteric stent was inserted. A follow-up CT of the urinary tract (Figure 5) performed six weeks later demonstrated improvement in the left retroperitoneal and abdominal collections, with no new collections identified. The enhancement of the left kidney improved, and the subcapsular collection remained stable.

## Discussion

Rupture of the ureter is a rare urological condition, and its underlying mechanisms are not well documented. One of the most common causes of ureteric rupture is ureteric stones [1]. In such cases, stone impaction or the downward movement of the calculus can lead to erosion and ulceration of the ureteric wall [2]. Besides stones, other factors such as malignancy, bladder outlet obstruction, idiopathic retroperitoneal fibrosis, and certain connective tissue disorders, like Klinefelter syndrome, which result in fibrotic changes, have also been linked to ureteric rupture [3].

Ureteric rupture lacks pathognomonic clinical features. Typically, patients present with non-specific symptoms, including generalized abdominal pain and tenderness, which may extend to renal angle tenderness on the side of the rupture [3]. Due to the ureter’s anatomy, symptoms may mimic conditions such as diverticulitis or appendicitis, as chemical irritation of the peritoneum occurs [4]. As a result, clinical signs may not provide sufficient diagnostic clues for ureteric rupture.

This was the case in our patient, who presented with a vague, non-specific history of feeling generally unwell, along with a productive cough and lethargy. Only upon physical examination were signs of an intra-abdominal pathology noted, including a distended abdomen and a palpable mass in the left upper quadrant extending into the epigastrium.

Given the non-specific nature of the clinical presentation, imaging becomes the key tool in diagnosing ureteric rupture. Intravenous contrast-enhanced CT imaging has the highest sensitivity for diagnosing ureteric rupture compared to other modalities, with CT urography being the first-line imaging modality [2-4]. CT urography is particularly useful for identifying the rupture site and assessing the degree of leakage based on contrast extravasation [2].

In our case, the diagnosis of left ureteric rupture was delayed, likely due to the repeated use of abdomen-pelvis CT scans. Initially, the suspected source of infection was considered to be intra-abdominal or related to general surgery, not urological causes. The first two CT scans were more indicative of IBD and a psoas muscle hematoma, respectively. It was only after the third abdomen-pelvis CT scan that a left distal ureteric calculus, ruptured ureter, and urinoma were identified.

Complications of ureteric rupture can be severe and life-threatening, as in our case. These may include urinoma, urosepsis, and abscess formation, either retroperitoneally or perinephrically [5,6]. Urinomas, a common complication, form as encapsulated urine collections outside the renal system, typically in the retroperitoneum [7]. If left undiagnosed or untreated, urinomas can lead to urinary peritonitis, renal atrophy, hypertension, and renal failure [7].

Given the potential severity of these complications, prompt treatment is essential and should be individualized for each patient. The literature generally favors minimally invasive procedures, such as percutaneous drainage (nephrostomy) and double-J stent insertion, as was done in our case [8-11]. Other treatment options include open surgery or conservative management with antibiotics and analgesia for stable patients [11,12].

Our patient also had a diagnosis of fragile X syndrome, a genetic disorder characterized by a deficiency or absence of the fragile X mental retardation 1 protein (FMR1) [13]. FMR1 plays a crucial role in translating mRNAs essential for the development of synaptic connections. The absence or reduction of FMR1 leads to unbalanced neuronal circuits, which manifests as intellectual disability, hyperactivity, autism spectrum disorder, impulsivity, and language development issues [13]. Although there is no known association between fragile X syndrome and ureteric rupture, it is important to note that patients with fragile X syndrome, especially adolescents and teenagers, may struggle to communicate symptoms such as pain [14].

In the present case, this contributed to the vague, non-specific history initially provided, which did not highlight any abdominal complaints. It was only through further investigation, based on a history provided by her mother, that symptoms related to her respiratory system, such as a productive cough with green sputum, were identified. This initially led to an investigation for sepsis of unknown origin. Delays in reaching a diagnosis were compounded by repeated abdomen-pelvis CT scans being requested instead of a CT urogram, the imaging modality of choice for diagnosing ureteric rupture.

## Conclusions

Due to its non-specific presentation, spontaneous ureteric rupture can be overlooked, leading to a focus on other intra-abdominal pathologies. A high index of clinical suspicion is needed to make an early diagnosis, which is crucial for better patient outcomes and management of complications. Managing patients with co-morbidities of fragile X syndrome or developmental delay requires special attention to be paid to their history, initial presentation, and examination findings and to have a low clinical threshold for treatment escalation if they deteriorate quickly. Hence, there is an increased need for awareness of spontaneous ureteric rupture in terms of recognition, diagnosis, and management, especially in patients with urinary lithiasis.
